# Protective Effects of Tualang Honey against Oxidative Stress and Anxiety-Like Behaviour in Stressed Ovariectomized Rats

**DOI:** 10.1155/2014/521065

**Published:** 2014-09-09

**Authors:** Badriya Al-Rahbi, Rahimah Zakaria, Zahiruddin Othman, Asma' Hassan, Asma Hayati Ahmad

**Affiliations:** ^1^Institute of Health Sciences, Muscat, Ruwi, P.O. Box 3720, Code 112, Oman; ^2^Department of Physiology, School of Medical Sciences, Universiti Sains Malaysia, 16150 Kubang Kerian, Malaysia; ^3^Department of Psychiatry, School of Medical Sciences, Universiti Sains Malaysia, 16150 Kubang Kerian, Malaysia; ^4^Department of Anatomy, School of Medical Sciences, Universiti Sains Malaysia, 16150 Kubang Kerian, Malaysia

## Abstract

The present study aims to evaluate the antioxidant and anxiolytic-like effect of Tualang honey in stressed ovariectomized (OVX) rats. The animals were divided into; (i) nonstressed sham-operated control rats, (ii) sham-operated control rats exposed to stress, (iii) nonstressed OVX rats, (iv) OVX rats exposed to stress, (v) OVX rats exposed to stress and treated with 17 *β*-oestradiol (E2) (20 *μ*g daily, sc), and (vi) OVX rats exposed to stress and treated with Tualang honey (0.2 g/kg body weight, orally). The open field test was used to evaluate the anxiety-like behaviour and ELISA kits were used to measure oxidant/antioxidant status of the brain homogenates. The result showed that anxiety-like behavior was significantly increased in stressed OVX compared to other groups, and administering either E2 or Tualang honey significantly decreased anxiety-like behaviour in stressed OVX rats. The levels of malondialdehyde (MDA) and protein carbonyl (PCO) were significantly decreased while the levels/activities of superoxide dismutase (SOD), glutathione S-transferases (GST), glutathione peroxidase (GPx), and glutathione reductase (GR) were significantly increased in the brain homogenates of treated stressed OVX groups compared to untreated stressed OVX. In conclusion, Tualang honey has protective effects against brain oxidative stress and may be useful alternative anxiolytic agent especially for postmenopausal women.

## 1. Introduction

Oxidative stress is defined as an imbalance between increased reactive oxygen species (ROS) production and inadequate antioxidant defense, a condition characterised by an overload in oxidants, which may culminate in cellular dysfunction [1]. Previous studies have demonstrated that oxidative stress is involved in aging and age-related processes that often accompany menopause [2–4].

The involvement of oxidative stress in neurodegenerative [5, 6] and psychiatric disorders [7], including anxiety [8] has gained particular interest, as the brain is considered especially sensitive to oxidative damage for a number of reasons [5]. The brain consumes considerable amount of oxygen and therefore produces comparatively large amount of free radical by-products, while having relatively modest antioxidant defenses. In addition, the brain is rich in lipid substrates for oxidation, as well as iron and copper ions that catalyse free radical reactions [5].

Mitochondrial dysfunction, inflammation, alterations in glutamate or gamma-aminobutyric acid (GABA) signalling, and inhibition of neurogenesis may contribute to increased oxidative stress, resulting in damage to cellular macromolecules. Eventually, the consequences are increased apoptosis, neuronal degeneration, and brain damage, leading to the manifestation of neuropsychiatric illnesses in susceptible individuals [9].

Previous studies observe a close relationship between oxidative stress and anxiety in both human patients suffering from anxiety disorders (obsessive-compulsive and panic disorders), and humans and animals displaying high trait anxiety [10, 11]. Studies have also focused on the link between redox status and normal anxiety, as well as on the possible causal relationship between cellular oxidative stress and emotional stress [12, 13].

Stress and oestrogen (E2) deficiency are the major contributors in increasing an individual's susceptibility to oxidative stress and affective disorders. Recently, our research group reported increased anxiety-like behavior in stressed ovariectomized (OVX) rats compared with nonstressed OVX or stressed sham-operated controls [14]. It was suggested that high circulating corticosterone acts synergistically with low circulating E2 to exert negative effects on the mood.

Studies have reported the beneficial effects of E2 [15–18] and dietary antioxidants including honey as therapeutic or preventive approach to health and mood disorders [19]. Malaysian Tualang honey is a pure, wild multifloral honey produced by Asian rock bees,* Apis dorsata* [20]. The bees build immense honeycombs on branches of very tall Tualang trees (*Koompassia excelsa*) in the Rain Forest of Northern Peninsular Malaysia [21]. Tualang honey is reported to have the highest antiradical activity compared to Gelam honey, Indian forest honey, and Pineapple honey [22]. Honey has been shown to reduce oxidative stress in animals [21, 23–25]. Study by our research group revealed that Tualang honey reduces oxidative stress in postmenopausal women [26]. Based on the above information, we hypothesised that Tualang honey, through its antioxidant effect, modulates anxiety-like behavior in stressed OVX rats.

The aims of this study were to evaluate the effects of Tualang honey supplementation on anxiety-like behavior and the brain antioxidant/oxidant system and to compare them with E2 treatment in stressed OVX rats.

## 2. Materials and Methods

### 2.1. Animals

Sixty adult females and 3 adult males Sprague-Dawley rats aged approximately 8 weeks old with body weight of 200 ± 20 g were obtained from the Animal Research and Service Centre (ARASC), Universiti Sains Malaysia (USM), Malaysia. All rats were housed in polypropylene cages (32 × 24 × 16 cm), exposed to 12 hr light-dark cycles, maintained at a room temperature of 23°C, and provided with free access to food and water. The cage bedding was changed every two days. The experimental protocol was approved by the Research and Animal Ethics Committee, USM.

The rats were randomly divided into six groups (*n* = 10 per group) as follows: (i) nonstressed sham-operated control rats; (ii) sham-operated control rats exposed to stress; (iii) nonstressed and OVX rats; (iv) OVX rats exposed to stress; (v) OVX rats exposed to stress and treated with 17 *β*-E2 (20 *μ*g daily, sc), and (vi) OVX rats exposed to stress and treated with Tualang honey (0.2 g/kg body weight, orally).

### 2.2. Surgical Procedure

Ovariectomy was performed under general anaesthesia following an intraperitoneal injection of 60 mg/kg of ketamine and 5 mg/kg of xylazine. The dorsal lumbar fur was shaved and cleaned with chlorhexidine scrub and 70% ethanol. A small 2 cm midline incision was made on the dorsal area over the 3rd to the 5th lumbar vertebrae. The ovarian fat pad was gently grasped using forceps and the ovary was exposed and removed. Following bilateral ovariectomy, the peritoneal cavity and skin were closed using absorbable sutures. The entire procedure was performed using aseptic technique. After the operation, animals were maintained under a heat lamp for one hour to avoid hypothermia. Sham-operated rats underwent a similar procedure, but without resection of the ovaries. Following surgery, animals were housed separately in clean cages for 10 days to avoid any interactions that might cause bleeding or poor healing. After 10 days, animals were then housed in a group of three per cage for two months.

### 2.3. Animal Treatment

The rats were treated with either 20 *μ*g/day 17*β*-oestradiol (Cayman Chemical, Ann Arbor, MI, USA) in 2.5 *μ*L corn oil injected subcutaneously [27] or 0.2 g/kg body weight/day of Tualang honey (Agro Mas, Federal Agricultural Marketing Authority (FAMA), Mergong, Kedah, Malaysia) diluted with 1 mL of distilled water and administered orally by gavage [28]. The treatments were started three days prior to stress procedures and continued throughout the 15 days of stress procedures.

### 2.4. Social Instability Stress Procedure

The social instability stress procedure was conducted two months after ovariectomy [29–32]. The procedure consisted of alternate isolation and crowding phases for 15 days as previously described [33]. The experiment started and ended with an isolation phase, with each phase lasting 24 hrs. Eight rats (three males and five females) were held per cage for the crowding phase. The initial 30 min of each crowding phase was videotaped. Biting attacks and dominant postures while fighting for food were counted [34, 35].

### 2.5. Behavioural Assessment

The open field test (OFT) was performed after the social instability stress procedure. The test was performed during the dark phase in a ventilated and soundproofed room that was maintained at a constant temperature of 23°C. The lighting intensity was maintained at a constant lux throughout the procedure with two 150-watt lamps.

The OFT arena consisted of a 35 cm high transparent plastic wall and a floor with a surface area of 120 × 120 cm^2^. White lines were drawn on the floor dividing the surface into 16 equal squares [36]. Three fluorescent lights provided diffuse overhead illumination. The animals were tested in the quiet room, and locomotive activity over a 5 min period was recorded using a digital camcorder placed at a control panel stationed 5 m from the testing apparatus for behavioural analysis later. Between each rat, the apparatus was cleaned with 70% alcohol to eliminate possible bias due to the odour that could have been left by the previous subject.

Recorded video was later scored by an experienced observer who was blinded to the condition of the animals. The mean number or duration of the following parameters was scored: (i) movement including rearing events (number of vertical activity), freezing time (seconds), and grooming time (seconds); (ii) locomotive activity, that is, time spent crossing the line (seconds); (iii) autonomic nervous system responses including defaecation (number of faecal boli) and number of face-washing events.

### 2.6. Blood Collections

The animals were sacrificed by decapitation immediately after the OFT. A total of 10 mL of blood was collected immediately via cardiac puncture. All blood samples were left to clot for 2 hours prior to centrifugation for 15 min at 4000 rpm (EBA 21, Hettich GmbH & Co. KG, Tuttlingen, Germany). Approximately 3 mL of serum was collected and stored at –20°C until assay.

### 2.7. Estimation of Serum Oestradiol and Corticosterone Levels

Serum oestradiol and corticosterone levels were measured using a specific ELISA kit (Creative Diagnostics, Shirley, NY, USA) according to the manufacturer's instructions. Briefly, 100 *μ*L of serum sample was added to each well followed by 100 *μ*L of enzyme-labelled oestradiol/corticosterone. The plate was incubated at 37°C for 90 min. Following incubation, the wells were carefully washed and 100 *μ*L of biotin-antibody working solution was added to each well and then incubated at 37°C for 60 min. Following three washes, 100 *μ*L of HRP was added to each well and then incubated at 37°C for 30 min. Next, 100 *μ*L of TMB Reagent was added to each well and then incubated at room temperature for 20 min, which resulted in a colour change. The colour change was stopped with the addition of 100 *μ*L of stop solution. The absorbance was then measured at 450 nm using a spectrophotometer (Thermo Fisher Scientific Inc. Waltham, MA, USA).

### 2.8. Preparation of Brain Homogenate

Brains from each group were quickly removed and carefully dissected in ice-cold saline to separate the brain hemispheres. The isolated hemispheres were then weighed and a homogenate (10% w/v) was prepared from the left hemisphere in ice-cold 0.1M phosphate-buffered saline (PBS, pH 7.4), whereas the right hemisphere was kept for histological study. The homogenates were then centrifuged with speed centrifugation (10,000 ×g) for 10 min. The samples were kept at −80°C until assay.

### 2.9. Assay of Antioxidant and Oxidative Stress Biomarkers

Commercially available kits were used to determine the level of superoxide dismutase (SOD), glutathione peroxidase (GPx) activity, glutathione reductase (GR) activity, glutathione S-transferases (GST), malondialdehyde (MDA), and total antioxidant capacity (TAC) (Northwest Life Science Specialties, Vancouver, USA) as well as the levels of catalase (CAT) and carbonyl proteins (PCO) (Cayman Chemical, Ann Arbor, MI, USA) in the brain homogenate. The total protein concentration was detected based on the Bradford method [37].

### 2.10. Statistical Analysis

Data were analysed using Predictive Analytics Software (PASW) version 20.0. Two-way analyses of variance (ANOVA) was utilised to examine the effects of stress (stress versus no stress) and surgery (sham-operated versus OVX) on anxiety-like behaviour. One-way ANOVA followed by post-hoc Tukey test was utilised to evaluate the effects of TH and oestradiol on anxiety-like behaviour, stress hormone level, and brain oxidative stress level/activity. Data were presented as the mean ± standard error of means (SEM). Probability values of less than 5% (*P* < 0.05) were considered statistically significant.

## 3. Results

### 3.1. Anxiety-Like Behaviour

The two-way ANOVA results on the effects of surgery and stress on anxiety-like behavior are summarised in Table 1. There was a significant main effect of stress on most of the anxiety-like behavior such as the total rearing number, grooming, time spent crossing the lines, freezing time, and number of boli. The change in anxiety-like behaviors was significantly higher in the stress than in the nonstressed rats.

Our data also indicated a significant main effect of surgery on most of the anxiety-like behaviors such as total rearing number, grooming, time spent crossing the lines, freezing time, and number of boli. The change in anxiety-like behaviors was significantly greater in OVX rats than in sham-operated control rats.

There was significant interaction between stress and OVX on most of the anxiety-like behaviors such as total rearing number, grooming, time spent crossing the lines, freezing time, and number of boli, indicating that sham-operated control and OVX rats were affected differently by stress. Most of the anxiety-like behavior was significantly increased following stress.

**Figure 1:**
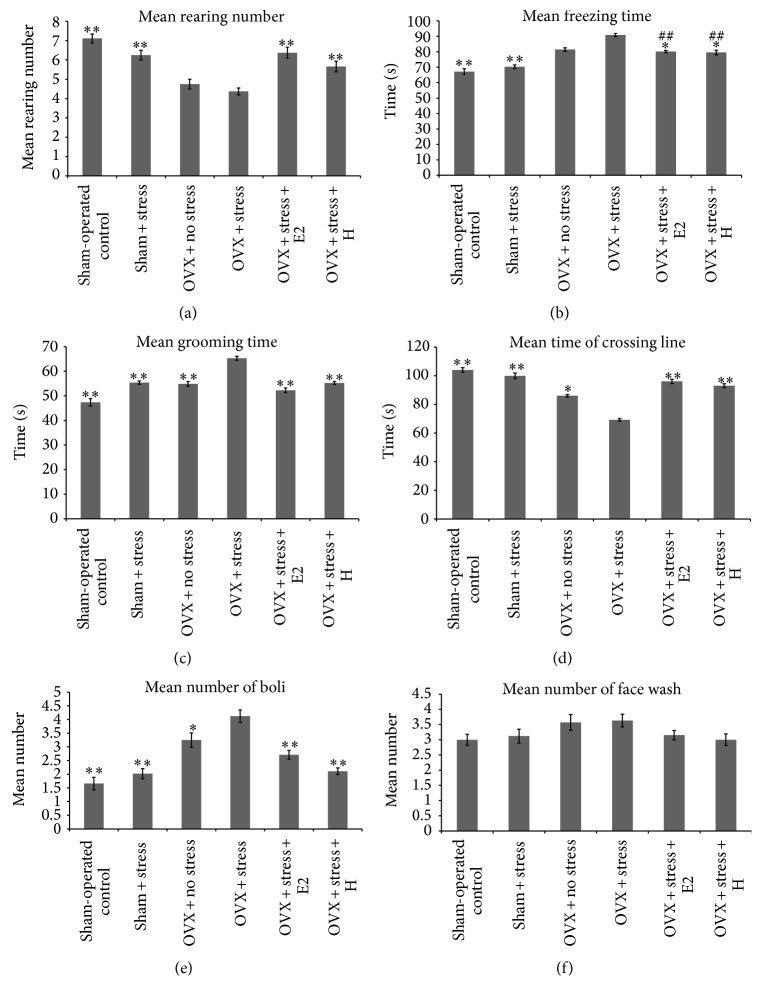
Effects of Tualang honey and E2 on anxiety-like behaviour. (a) Mean rearing number was significantly increased in stressed OVX rats treated with either E2 or Tualang honey compared with untreated stressed OVX. (b) Mean freezing time (s) was significantly decreased in stressed OVX rats treated with either E2 or Tualang honey compared with untreated stressed OVX. (c) Mean grooming time (s) was significantly decreased in stressed OVX rats treated with either E2 or Tualang honey compared with untreated stressed OVX. (d) Mean duration of crossing line (s) was significantly increased in stressed OVX rats treated with either E2 or Tualang honey compared with untreated stressed OVX. (e) Mean number of boli rearing number was significantly decreased in stressed OVX rats treated with either E2 or Tualang honey compared with untreated stressed OVX. (f) Mean number of face washing was not significantly different between the experimental groups. The data are shown as means ± S.E.M. ∗*P* < 0.05 ∗∗*P* < 0.001 denote significantly different values from stressed OVX group. ^#^ *P* < 0.05 ^##^ *P* < 0.001 denote significantly different values from nonstressed sham-operated control group.

The one-way ANOVA revealed that the mean number of rearing events (*F*(5, 54) = 13.72,* P* < 0.001), freezing time (*F*(5, 54) = 35.27,* P* < 0.001), grooming time (*F*(5, 54) = 37.87,* P* < 0.001), time spent crossing the line (*F*(5, 54) = 16.73,* P* < 0.001), and the number of boli (*F*(5, 54) = 18.49,* P* < 0.001) were significantly different between the groups. However, there was no significant difference (*P* < 0.05) in the mean number of face washing between the groups as shown in Figure 1.

The post hoc analysis revealed significant increase in the mean number of rearing events and time spent crossing the line, and significant decrease in the mean grooming time and the number of boli in stressed OVX treated with either E2 or Tualang honey when compared to untreated stressed OVX rats. Both E2 and Tualang honey significantly increased the mean number of rearing events and time spent crossing the lines, and significantly decreased the mean grooming time and the number of boli of treated stressed OVX comparable to that of sham-operated control rats.

The post hoc analysis showed that the mean freezing time was significantly decreased in stressed OVX treated with either E2 or Tualang honey (*P* < 0.05) compared to untreated stressed OVX. Both E2 and Tualang honey reduced the mean freezing time of treated stressed OVX comparable to that of nonstressed OVX but the values remained higher than that of sham-operated control rats.

### 3.2. Serum Oestradiol and Corticosterone Levels

The serum level of oestradiol was significantly higher in sham-operated when compared with OVX groups except for OVX rats treated with 17 *β*-oestradiol (230.10 ± 11.29 versus 81.80 ± 2.91 pg/mL;* P* < 0.05). Meanwhile, serum corticosterone levels were significantly lowered in nonstressed compared to stressed rats (4611.00 ± 169.43 versus 5661.90 ± 163.95 pg/mL;* P* < 0.05).

### 3.3. Brain Oxidant/Antioxidant Status

**Figure 2:**
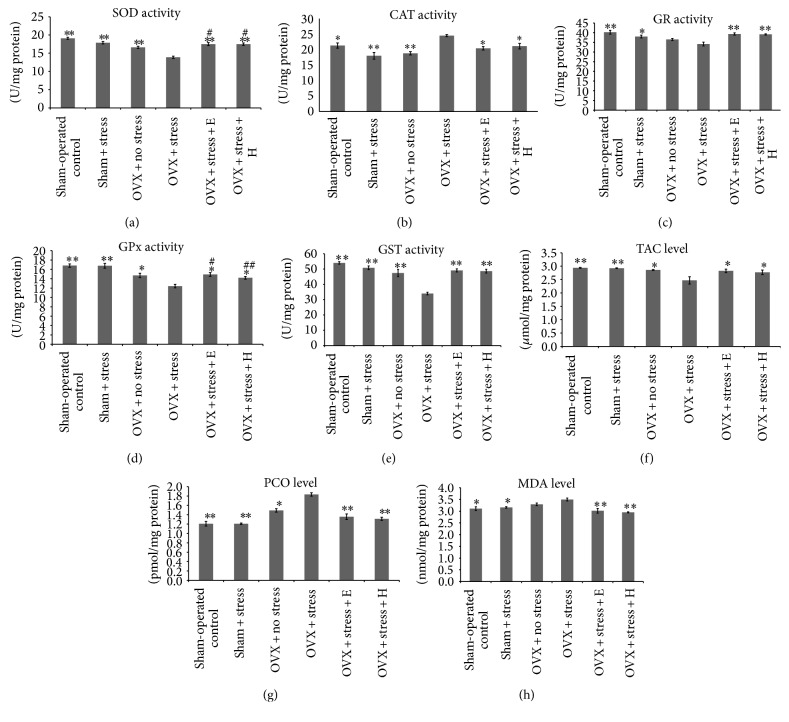
Effects of Tualang honey and E2 on brain oxidant/antioxidant level/activity. (a) Mean SOD activity was significantly increased in stressed OVX rats treated with either E2 or Tualang honey compared with untreated stressed OVX. (b) Mean CAT activity was significantly decreased in stressed OVX rats treated with either E2 or Tualang honey compared with untreated stressed OVX. (c) Mean GR activity was significantly increased in stressed OVX rats treated with either E2 or Tualang honey compared with untreated stressed OVX. (d) Mean GPx activity was significantly increased in stressed OVX rats treated with either E2 or Tualang honey compared with untreated stressed OVX. (e) Mean GST activity was significantly increased in stressed OVX rats treated with either E2 or Tualang honey compared with untreated stressed OVX. (f) Mean TAC level was significantly increased in stressed OVX rats treated with either E2 or Tualang honey compared with untreated stressed OVX. (g) Mean PCO level was significantly decreased in stressed OVX rats treated with either E2 or Tualang honey compared with untreated stressed OVX. (h) Mean MDA level was significantly decreased in stressed OVX rats treated with either E2 or Tualang honey compared with untreated stressed OVX. The data are shown as means ± S.E.M. ∗*P* < 0.05 ∗∗*P* < 0.001 denote significantly different values from stressed OVX group. ^#^ *P* < 0.05 ^##^ *P* < 0.001 denote significantly different values from nonstressed sham-operated control group.

The present study showed significant differences among the groups in the mean activity/level of SOD (*F*(5, 54) = 26.75,* P* < 0.001), CAT (*F*(5, 54) = 9.17,* P* < 0.001), GR (*F*(5, 54) = 9.61,* P* < 0.05), GPx (*F*(5, 54) = 16.75,* P* < 0.001), GST (*F*(5, 54) = 26.51,* P* < 0.001), TAC (*F*(5, 54) = 5.95,* P* < 0.001), MDA (*F*(5, 56) = 9.55,* P* < 0.001), and PCO (*F*(5, 56) = 33.08,* P* < 0.001) as shown in Figure 2.

Both E2 and Tualang honey increased the mean activity/level of GR, GST, and TAC and reduced the mean activity/level of CAT, PCO, and MDA comparable to that of sham-operated control rats. The mean activity/level of SOD and GPx of treated stressed OVX rats was also significantly increased but the values remained lower than that of sham-operated control rats.

## 4. Discussion

We found that the anxiety-like behavior was significantly increased in stressed rats compared to nonstressed rats. Our results were also consistent with those of previous animal studies that reported decreased activity in an open field following predator (cat) exposure stress [38] and chronic restraint stress [39, 40]. Previous studies also revealed that chronic stress or chronically elevated levels of glucocorticoids exert detrimental effects on the brain and behavior [41, 42]. However, our data were inconsistent with previous studies that found increased locomotion and reduced defecation scores [43, 44] or no change [45] following chronic social isolation stress. This inconsistency may be attributed to methodological differences. It is possible that the crowding phase and not the isolation phase contributes to anxiety-like behaviors.

The present study also found increased anxiety-like behavior in OVX rats compared to sham-operated controls. This finding was in parallel with previous animal studies [46–48]. Galeeva and Tuohimaa [46] found that exploration of open arms is reduced in diestrous females, suggesting that female mice are more anxious when their E2 levels are low. Previous studies revealed that OVX rats exhibit high anxiety-like behavior compared to sham-control rats [47, 49]. A recent study by Daendee et al. [48] examined anxiety-like behavior with elevated T-maze (ETM) and open field 7, 14, 21, and 28 days after OVX. They found increased anxiety as shown by increase in inhibitory avoidance latency in the ETM with significant effect at day 21 and even higher at day 28. Human studies also documented that women are 1.5 times more likely than men to exhibit social anxiety-like disorder. This vulnerability to anxiety-like disorders in women has been attributed to low levels of E2 and progestin [50–52].

Interestingly, a significant decrease in the overall anxiety-like behavior was noted in stressed OVX rats treated with either E2 or Tualang honey for 18 days compared to untreated rats. The overall improvements in anxiety-like behavior are comparable with that of nonstressed sham-operated rats and are in line with those of previous animal studies [49, 53, 54]. Mora et al. [55] reported greater percentage of time spent in the open arms indicating reduced anxiety-like behavior in E2 benzoate-treated OVX rats compared with vehicle-treated female rats. Furthermore, clinical studies also reported that E2 replacement therapy in postmenopausal women is associated with improved anxiety and depression symptoms [56–58].

The effects of E2, however, have been reported to be dose and duration dependent. Very low or high dosages of E2, which is not expected to significantly increase the circulating E2 concentrations to the levels observed in naturally receptive rats, result in only slight or no decrease in anxiety-like behavior [59, 60]. In addition, chronic E2 treatment is necessary to elicit more stable effects on anxiety-like behavior because various doses of acute E2 treatment have been shown not to affect anxiety-like behavior [59]. Morgan and Pfaff [44] suggested that the increased anxiety-like behavior in OVX mice treated with 25 *μ*g/day of E2 benzoate for 5 weeks is due to the increase in arousal process.

Antioxidant properties of E2 have been suggested to improve hippocampal and cortical histology by reducing brain lipid peroxidation [61]. Our group recently reported that E2 treatment in stressed OVX rats enhanced neuronal proliferation of hippocampal CA2, CA3, and DG regions compared to untreated stressed OVX rats [62]. In the present study, we found higher activities of SOD, GST, GPx, and GR in the brain homogenates of E2-treated group compared with untreated stressed OVX rats and these findings possibly explain the improvement in hippocampus morphology in our previous reports [62]. Chronic E2 treatment triggers significant increase in GST and GR activities in the aorta of OVX rabbit compared with the untreated group [63]. Increased SOD and GPX activity, decreased MDA, and reduced PCO were also observed in OVX rats receiving chronic E2 treatment [64–66]. A recent study by Ceravolo et al. [67] also found that chronic conjugate equine E2 therapy significantly enhances CAT expression in OVX rats compared to untreated rats. In addition, clinical studies noted higher CAT [26], GPx [26], SOD [68], and TAC [69] activities/levels in postmenopausal women treated with chronic HRT.

The antioxidative activities of E2 possibly act via two different mechanisms. The first mechanism is by using the hydroxyphenolic structure of E2 as an important free-radical scavenger [69] independent of the ER activation [61, 70]. Modification or removal of the phenolic group blocks the antioxidant activities of E2 [71]. The second mechanism is related to the influence of E2 on endogenous antioxidative enzyme systems [68]. E2 is highly lipid soluble and largely resides in the membrane component of cells [72], where they are ideally suited to affect the oxidation of unsaturated bonds in phospholipids. This membrane localisation allows E2 to interact synergistically with abundant antioxidants, such as glutathione [73, 74]. Another study revealed that E2 can prevent lipid peroxidation by sacrificing itself, resulting in a quinol product [75].

However, our data were inconsistent with that of a previous clinical study which showed no significant difference in SOD level between HRT-treated and untreated postmenopausal women [76], as well as an animal study that showed reduced GPx activities in both plasma and brain tissues of OVX rats treated with chronic E2 combinations [77]. These discrepancies could possibly be due to E2 effects being less prominent at older age group or act differently in human and animal.

Interestingly, our study also revealed that chronic Tualang honey supplementation in stressed OVX rats produced anxiolytic effects comparable to E2. Our finding supports an earlier study showing decreased anxiety-like behavior in rats fed with 10%, 20%, and 40% honey compared with those fed with either sucrose or sugar-free diets [19]. A recent study conducted by Oyekunle et al. [78] also found that female rats fed with 20% and 40% bee honey concentrations demonstrate anxiolytic-like effects that are attributed to the antioxidant properties of pure bee honey. In addition, other antioxidant compounds such as rosmarinic acid, zingicomb, chlorogenic acid, and* Withania somnifera *are able to reduce anxiety-like behavior in rats and mice [79–82]. Bhattacharya et al. [79] suggested that* Withania somnifera,* an antioxidant diet, increases the activities of antioxidant enzymes in rat brain frontal cortex and striatum and thus reduces anxiety-like behaviour. It is also possible to suggest that Tualang honey reduces the brain oxidative stress that in turn modulates the brain 5-HT system and reduces anxiety-like behaviour.

In conclusion, Tualang honey supplementation has protective effects against brain oxidative stress and anxiety-like behavior comparable to E2 treatment in stressed OVX rats. This effect possibly works through improvement in brain oxidant/antioxidant status and modulation of the brain 5-HT system. We therefore propose that Tualang honey is an alternative anxiolytic agent especially for postmenopausal women. However, further confirmatory studies are required to determine its usage as an anxiolytic agent in anxiety disorders and its clinical effective dose.

## Figures and Tables

**Table 1:** Effects of OVX and stress on anxiety-like behaviour.

		Duration (seconds)			Number	
	Grooming	Crossing the lines	Freezing	Face washing	Total rearing	Boli
Sham-operated control						
No stress	47.37 ± 1.48	104.01 ± 1.6	67.12 ± 1.89	3.00 ± 0.18	7.11 ± 0.93	1.66 ± 0.23
Stress	55.37 ± 0.68	99.89 ± 1.98	70.25 ± 1.22	3.12 ± 0.23	6.25 ± 0.65	2.02 ± 0.18
OVX						
No stress	54.87 ± 0.98	86.01 ± 0.85	81.37 ± 1.19	3.57 ± 0.26	4.75 ± 0.25	3.25 ± 0.26
Stress	65.32 ± 0.81	69.23 ± 0.98	90.81 ± 1.05	3.63 ± 0.21	4.37 ± 0.18	4.12 ± 0.23
Stress effect	*P* < 0.001	*P* < 0.001	*P* < 0.001	*P* > 0.05	*P* < 0.01	*P* < 0.01
OVX effect	*P* < 0.001	*P* < 0.001	*P* < 0.001	*P* > 0.05	*P* < 0.001	*P* < 0.001
Stress X OVX interaction	*P* < 0.01	*P* < 0.01	*P* < 0.01	*P* > 0.05	*P* < 0.05	*P* < 0.05

Note: Values are expressed as mean ± SEM. Two-way ANOVA were utilised to examine the effects of stress (stress versus no stress) and surgery (sham-operated versus OVX) on anxiety-like behaviour. *P* < 0.05 indicates significant difference.
